# A year like no other: introduction to a special issue on COVID-19 and pain

**DOI:** 10.1097/PR9.0000000000000915

**Published:** 2021-05-10

**Authors:** Michael C. Rowbotham, Lars Arendt-Nielsen

**Affiliations:** a Pain Management Center, University of California School of Medicine, San Francisco, CA, USA; b Treasurer, International Association for the Study of Pain (IASP), Washington, DC, USA; c Center for Neuroplasticity and Pain (CNAP), School of Medicine, Aalborg University, Aalborg, Denmark

As of the end of January 2021, one full year into the coronavirus pandemic, more than 100 million people around the world have been sickened, according to official counts. More than 2 million have died, and the virus has been detected in nearly every country. Major news outlets, such as the *New York Times*, publish a daily update with time trends for every country and all states (and their counties) in the United States.^17^

Countries that had emerged from the first wave with low case rates have experienced alarming increases. Much of Europe, after a devastating initial wave, experienced an even larger spike toward the end of 2020. Multiple countries have resumed lockdowns and curfews they found to be effective for reducing case rates.

Vaccinations began worldwide in mid-December with the Pfizer/BioNT and the Moderna vaccines, and earlier with the vaccines created in Russia and China. Alarm is growing about new highly infectious and rapidly spreading variants emerging from the United Kingdom, South Africa, Brazil, the United States, and elsewhere.^9^ Whether the new variants can overcome currently available vaccines, monoclonal antibody therapies, convalescent serum, and even immunity from previous SARS-CoV-2 infection is uncertain.

Since bursting into our daily lives, there has been a torrent of scientific articles on the SARS-CoV-2 virus, COVID-19 disease, and its impact. The pace of science has accelerated tremendously. A study covering just the period January 1 to June 30 found an estimated 23,634 unique published articles indexed on Web of Science and Scopus, and may soon exceed 60,000 articles.^16^ A primary vehicle of open science is the so-called “preprint server,” a place on the Internet where research teams can download their articles to be viewed, openly and publicly, free of charge, and without the time-consuming process of rigorous journal peer review. Intense media attention and blistering critical comments from other scientists may quickly follow, hence the term “crowd-sourced” peer review. The first trickle of postings from China began in January 2020 on the preprint server bioRxiv, founded in 2013 as a service of the Cold Spring Harbor Laboratory. By early September, more than 8,500 articles had appeared on bioRxiv and its sister site medRxiv. Thankfully, many prestigious medical journals and major news outlets have made COVID-19-related articles accessible free of charge to everyone.

Although the rapid dissemination of new findings is undoubtedly a blessing, the Surgisphere database scandal stunned the scientific community in 2020. Retractions by *The Lancet* and *The New England Journal of Medicine* followed, along with thoughtful calls for reviewing how science is conducted, published, and acted on.^13^

Understandably, the literature has concentrated on the SARS-CoV-2 virus and the COVID-19 disease itself and on the management of acute cases. Current evidence clearly demonstrates that patients with COVID-19 experience symptoms such as headache, dizziness, neuralgias (burning pain), neuropathies, and myalgias.^1,2^ Patients with COVID-19 admitted to an intensive care unit may develop severe “critical illness polyneuropathy,” perhaps with an incidence higher than other groups of ICU patients. Song and colleagues in China reported that patients hospitalised by COVID-19 infection presented with mild to moderate body pain resembling a pattern compatible with myalgias of musculoskeletal origin (COVID-19 viral-induced myalgias).^15^ Several studies report that about 36% of patients with COVID-19 infection have myalgias.^1,18^ The World Health Organization recognises that around 15% of patients with COVID-19 experience muscle pain (myalgia), joint pain (arthralgia), and headache as associated pain symptoms.^19^

Once the acute phase and need for hospitalisation abate, recovery may be protracted or incomplete.^11,18^ The incidence of long-term COVID-19-related pain is unclear. Millions more people will survive COVID-19 infection and the world should prepare for long-term pain and other sequelae in many. This should be a priority not only for pain researchers and clinicians but also for health care systems in general.

A recent article by Lawrence Wright, entitled *The Plague Year*, describes the chronology of the twists and turns faced by the CDC and government agencies around the world as they struggled to understand the virus, treat infected patients, and develop containment strategies.^20^ They rapidly realized how the SARS-CoV-2 virus is unusual among respiratory viruses. First, it directly infects the endothelial cells lining our blood vessels. When the virus invades them, instead of helping to protect the body from infection, their powerful chemical contents are discharged into the bloodstream to create widespread inflammation. Breaks and vessel irregularity can cause turbulent blood flow.

Second, COVID-19 leads to hypercoagulability with blood clot formation resulting in stroke and pulmonary emboli, even clotting within lung tissue. Third, although infectious diseases often kill people by triggering an excessive immune system response, COVID-19 disease was unusual in the breadth of bodily malfunctions. Some patients require kidney dialysis, suffer liver damage, insulin-dependent diabetes, cardiomyopathy, delirium and psychosis, strokes, and lasting nerve damage.

Many hypotheses have been proposed for explaining the presence of pain during and after COVID-19 infection, including increased levels of the proinflammatory cytokines, immune-mediated injury to central and peripheral pain systems, direct infection of neurons, spinal cord changes by ACE2 enzyme, vascular injury, and hypercoagulability.^5,14^ Mechanistically, elevated IL-6, IL-10, and TNF cytokine levels are associated with the development of muscle pain, making it highly plausible that a frequent post COVID-19 sequel will be persistent myalgic pain.^18^

In this special issue of *PAIN Reports*, 11 peer-reviewed articles focus on multiple aspects of COVID-19 and pain. Country-focused reports from Europe, North America, and China describe their experience of the initial wave of the pandemic, including how their health care systems were impacted and rapidly changed to meet the challenge of managing new pain while continuing to provide care for existing chronic pain patients.^3,4,6–8,10,12^ Unintended consequences of pandemic-deferred pain treatment and psychological consequences of activity restrictions on the experience of chronic pain have been universal. All the country reports in the collection describe the emergence of patients with long-term COVID-19 persistent pain, including pain related to long and arduous hospital stays. Telehealth came to the fore in many locations, and the group at McGill University in Montreal provide a thoughtful guide to effectively instituting telehealth approaches.^10^ Authors from Patient-Led Research for COVID-19 describe their evolution from an online support group of COVID-19 survivors into a large and robust group conducting large-scale patient-centric and patient-led research.^11^ Three articles focus on the basic and clinical science of COVID-19-related pain, neurological complications, and how the new pain syndromes compare to previously recognized post-viral pain and ME/CFS.^2,14,18^

The characteristics of the pandemic's second/third wave with respect to COVID-19 pain sequelae are beginning to be studied. A global effort focusing on pain sequelae in COVID-19 survivors is needed to ensure recognition and immediate action (counselling, diagnostics, and management) for those struggling with complex and underrecognised pain syndromes.

## Disclosures

The authors have no conflicts of interest to declare.
